# Kaposi’s Varicelliform Eruption in a Child with Atopic Dermatitis: A Case Report

**DOI:** 10.3390/reports9030275

**Published:** 2026-08-17

**Authors:** Alfonso Lendínez-Jurado, Ana García-Ruiz, Viviane Ruiz-Dassy

**Affiliations:** 1 UGC Churriana, Distrito Sanitario Málaga-Guadalhorce, 29009 Málaga, Spain; ana.garcia.ruiz.sspa@juntadeandalucia.es (A.G.-R.); mviviane.ruiz.sspa@juntadeandalucia.es (V.R.-D.); 2 IBIMA Plataforma BIONAND, Instituto de Investigación Biomédica de Málaga, 29590 Málaga, Spain

**Keywords:** eczema herpeticum, Kaposi varicelliform eruption, atopic dermatitis, herpes simplex virus, acyclovir, pediatric dermatology

## Abstract

**Background and Clinical Significance:** Eczema herpeticum (EH), or Kaposi’s varicelliform eruption, is a dermatologic emergency characterized by the abrupt onset of painful monomorphic vesiculopustules with potential for rapid dissemination. Atopic dermatitis (AD) is the main predisposing condition due to skin barrier dysfunction and impaired antiviral immunity. Early recognition is essential because delayed treatment may result in avoidable complications, including ocular involvement and systemic disease. Current recommendations emphasize immediate systemic acyclovir based on clinical suspicion, without awaiting laboratory confirmation. **Case Presentation:** A 4-year-old boy with moderate AD presented with a 6-day history of fever, malaise, and a rapidly progressive vesiculopustular eruption involving both eczematous and previously unaffected skin. The patient had a recent AD flare, molluscum contagiosum, and had initially received oral amoxicillin-clavulanate for presumed bacterial superinfection without improvement. Physical examination revealed widespread painful monomorphic umbilicated vesiculopustules with hemorrhagic crusts and mild bilateral conjunctival injection. Oral acyclovir was initiated within one hour of evaluation. Laboratory investigations showed mild inflammatory abnormalities without renal or hepatic involvement. Because lesional PCR was unavailable, complementary blood-based investigations were performed; HSV-1 IgM serology and blood PCR provided additional retrospective findings compatible with HSV-1 infection, while Gram stain and bacterial cultures were negative. Fever resolved within 24 h, no new lesions developed after day 3, and complete re-epithelialization was achieved after a 10-day course of acyclovir. **Conclusions:** This case highlights the importance of bedside recognition of eczema herpeticum in children with atopic dermatitis, particularly when painful monomorphic vesiculopustules are accompanied by fever and rapid dissemination. Early initiation of systemic acyclovir based on clinical suspicion remains the cornerstone of management. While PCR from vesicular lesions is the preferred diagnostic test when available, laboratory confirmation should not delay treatment. This report also illustrates common real-world challenges, including initial misdiagnosis as bacterial infection and limited access to optimal virological testing.

## 1. Introduction and Clinical Significance

Eczema herpeticum (EH), also known as Kaposi varicelliform eruption (KVE), is a viral dermatologic emergency characterized by the sudden eruption of monomorphic vesiculopustules with the potential for rapid systemic dissemination. Although classically attributed to herpes simplex virus type 1 (HSV-1), and less often HSV-2, cases caused by vaccinia virus, Coxsackie A6/A16, and varicella-zoster virus have also been documented, highlighting the broader pathogen spectrum associated with this clinical entity [1,2,3].

Atopic dermatitis (AD) continues to be the principal predisposing factor, with up to 3% of children with AD developing EH during the course of their disease [4]. Advances in immunology and barrier biology over the last decade have clarified that individuals with AD exhibit a complex triad of [5,6,7,8]:

- Structural skin barrier impairment—including filaggrin loss-of-function mutations, disrupted ceramide profiles, and impaired tight junctions;
- Immune dysregulation dominated by Th2 cytokines (IL-4, IL-13), impairing antiviral defenses;
- Microbial dysbiosis, especially Staphylococcus aureus dominance, which amplifies inflammation and compromises the cutaneous barrier.

Evidence from ex vivo infection models in recent years has demonstrated that IL-4/IL-13-driven inflammation alone is sufficient to permit epidermal penetration of HSV-1, even in previously healthy skin, simulating an AD-like phenotype [9]. This breakthrough has strengthened the mechanistic link between AD flares and HSV susceptibility.

EH typically presents with the abrupt onset of painful monomorphic vesicles, often showing central umbilication and hemorrhagic crusting. Fever, lymphadenopathy, malaise, and varying degrees of systemic involvement may be present. The disease can progress rapidly and lead to serious complications, including herpetic keratoconjunctivitis, encephalitis, sepsis, hepatic involvement, and multi-organ failure, particularly in children, infants, and immunocompromised individuals [10,11,12]. Ocular involvement is of particular concern because delayed diagnosis may result in corneal scarring and permanent visual impairment [13,14].

Recent pediatric series indicate that EH remains frequently misdiagnosed, often resulting in unnecessary antibiotic treatment and delays in antiviral therapy [15]. Current guidelines from the American Academy of Allergy, Asthma & Immunology (AAAAI) and the American College of Allergy, Asthma & Immunology (ACAAI) recommend prompt initiation of systemic acyclovir whenever EH is clinically suspected [16].

PCR from the base of a fresh vesicle remains the preferred diagnostic test when available, whereas blood HSV PCR should be reserved for selected severe cases or those with suspected systemic involvement [1,4,10,15]. Importantly, laboratory confirmation should not delay treatment [10,16].

Given these challenges, EH requires a high index of clinical suspicion, particularly in children. Early recognition, appropriate differential diagnosis, and prompt antiviral therapy remain essential to prevent morbidity and long-term sequelae.

## 2. Case Presentation

A 4-year-old boy with a known history of moderate AD since infancy, who had received all age-appropriate routine childhood vaccinations including two doses of varicella vaccine, was brought to the pediatric emergency department with a 6-day history of progressively worsening malaise, irritability, and cutaneous eruption. According to his parents, the illness began with intensely pruritic papulovesicular lesions initially confined to the right forearm, which rapidly disseminated to the arms, trunk, buttocks, and lower extremities over 48 h. The child developed high-grade fever peaking at 39.8 °C, persistent for four days, only partially responsive to paracetamol and ibuprofen. Progressive fatigue, decreased appetite, and disrupted sleep were also reported.

The boy had a recent diagnosis of molluscum contagiosum involving the trunk and upper extremities, treated with topical potassium hydroxide, and had been experiencing an AD flare with extensive excoriations. His primary care physician had empirically initiated oral amoxicillin–clavulanate for suspected bacterial superinfection, without clinical improvement. No recent contact with individuals with cold sores or varicella was reported, though the child attended kindergarten where viral illnesses were common.

**Figure 1:**
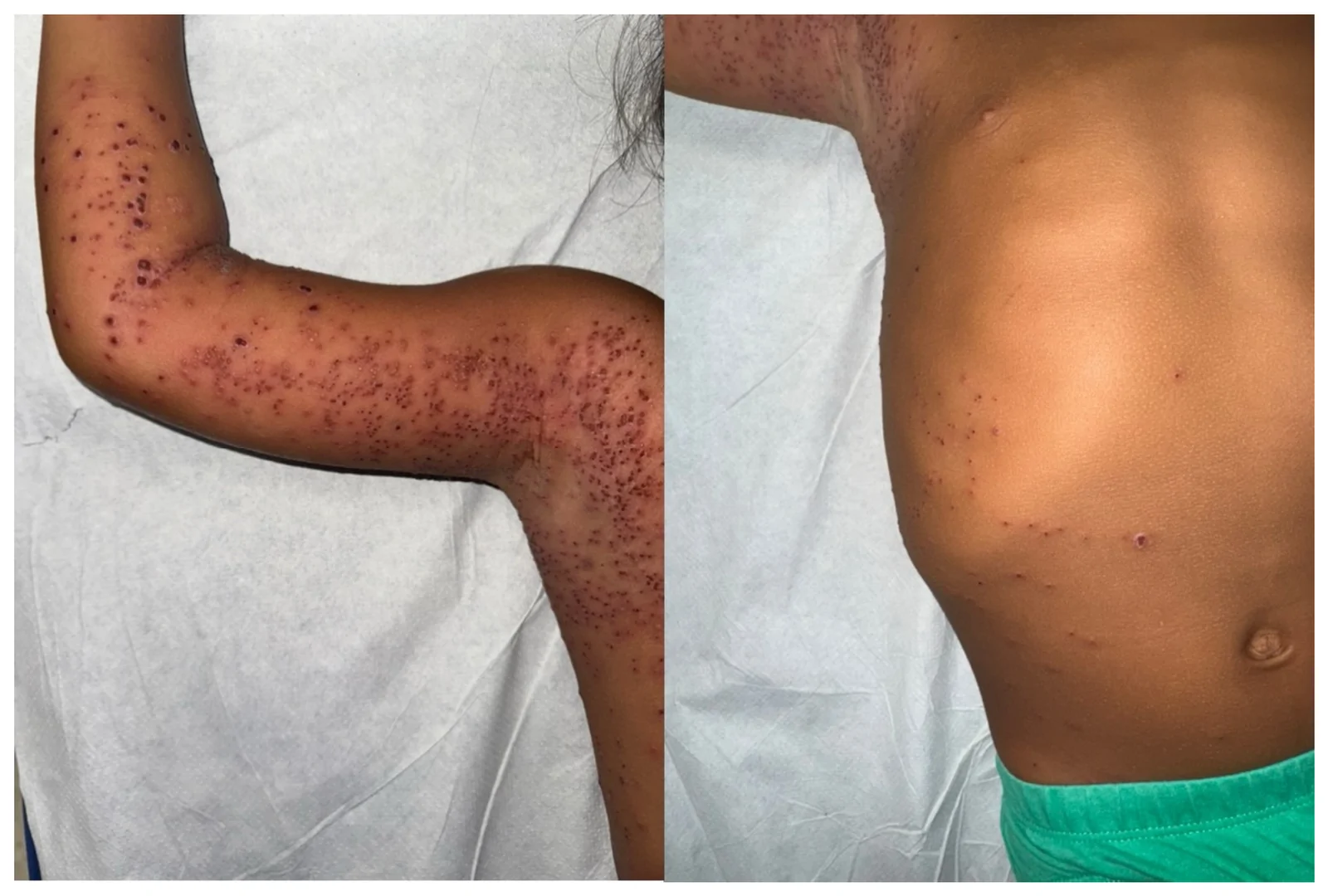
Extensive clusters of vesiculopustular lesions on the upper limb and axilla, with scattered lesions on the lateral thorax, consistent with disseminated Kaposi’s varicelliform eruption.

On examination, the patient appeared tired and irritable but was hemodynamically stable. Dermatologic inspection revealed a striking eruption of monomorphic vesiculopustules, several demonstrating central umbilication, distributed both on eczematous plaques and previously unaffected skin (Figure 1). Numerous lesions exhibited hemorrhagic crusting, and several had coalesced into erosive plaques. The axilla, neck, thorax, and flexural surfaces were prominently involved. Notably, the child had pain on palpation, a characteristic more suggestive of EH than impetiginized eczema.

Mild bilateral conjunctival injection was present, though without photophobia, ocular pain, visual complaints, or discharge. No periocular vesicles were observed. Ophthalmologic consultation was not available at the time of assessment. The patient was therefore closely monitored clinically, and no signs suggestive of keratitis or progressive ocular involvement developed during follow-up. Cervical lymphadenopathy was palpable but non-tender. The remainder of the systemic examination was unremarkable, and signs of dehydration were absent (Table 1).

Given the high clinical suspicion for EH, oral acyclovir at 20 mg/kg every 6 h was initiated within the first hour of evaluation. Routine laboratory investigations were performed during the emergency department assessment and are summarized in Table 2. Because virological testing of lesion samples was not available, complementary blood-based investigations were obtained. Bacterial cultures were negative. HSV-1 serology and blood PCR subsequently yielded retrospective findings compatible with HSV-1 infection. Over the following 24 h, the child’s fever subsided and the lesions began to crust.

**Figure 2:**
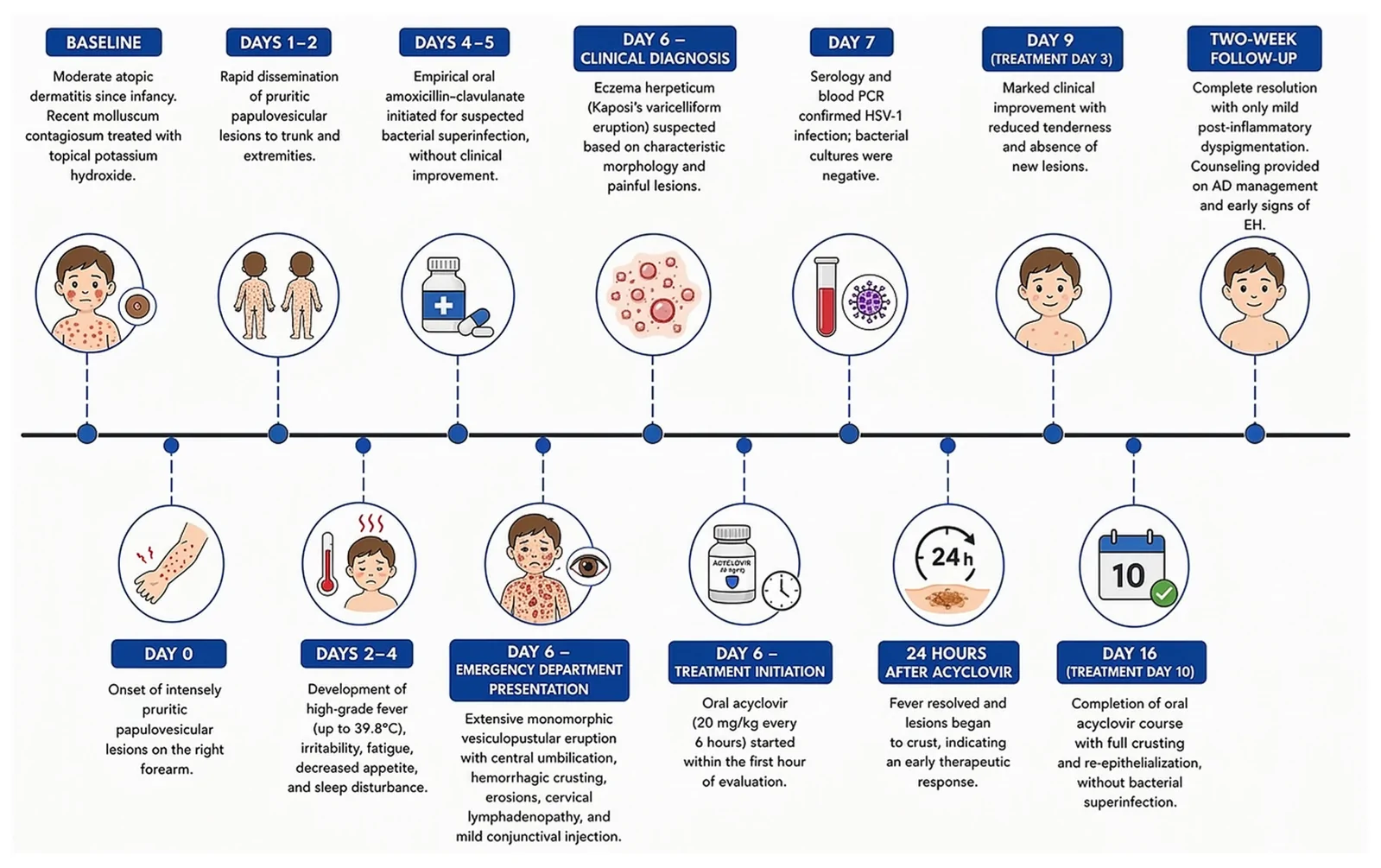
Clinical timeline of the reported case.

By day 3, significant improvement was noted, with reduced tenderness and no new lesions. The child completed a 10-day course of oral acyclovir administered under caregiver supervision, achieving full crusting and re-epithelialization without bacterial superinfection. Caregivers received instructions regarding treatment adherence, recognition of warning signs requiring reassessment, and appropriate skin care measures. A follow-up visit two weeks later showed complete resolution of the eruption with only mild post-inflammatory dyspigmentation. Additional counseling was provided regarding long-term AD management and early recognition of future episodes of eczema herpeticum. The chronological sequence of clinical manifestations, diagnostic investigations, treatment, and follow-up is summarized in Figure 2.

## 3. Discussion

EH represents a convergence between viral virulence and host cutaneous vulnerability, particularly in the context of AD. In our patient—a 4-year-old with moderate AD—several well-recognized vulnerability factors were present at presentation: an ongoing AD flare with extensive excoriations (mechanical barrier loss) and a recent history of molluscum contagiosum, which commonly co-exists with AD flares and reflects impaired cutaneous antiviral defenses. These findings are consistent with the recognized barrier dysfunction of AD, which facilitates viral entry and dissemination [1,4]. The child’s rapidly spreading, painful, monomorphic vesiculopustular eruption on both eczematous and previously unaffected skin fits classic EH biology, in which barrier failure and pain/dissemination are prominent. Although the clinical phenotype of eczema herpeticum is well established, this case illustrates several challenges that remain highly relevant in daily pediatric practice, including initial treatment as presumed bacterial infection, limited access to lesional PCR, ocular findings at presentation, and the need to initiate antiviral therapy before microbiological confirmation.

Beyond structural failure, type-2 immune skewing is central to EH susceptibility. Th2 cytokines (IL-4, IL-13) suppress keratinocyte antiviral interferon responses. Experimental evidence indicates that IL-4/IL-13-driven inflammation facilitates HSV-1 penetration of the epidermis and impairs local antiviral defense mechanisms, providing a mechanistic link between AD flares and acute eczema herpeticum [9]. In our patient, this pathogenic background may help explain the rapid dissemination of lesions observed over a 48 h period.

Additionally, differential diagnosis was a key component of the initial assessment. The presence of painful, monomorphic, umbilicated vesicles with hemorrhagic crusts and the very rapid centrifugal spread pointed strongly to EH rather than impetiginized eczema. Nevertheless, in young children with AD, eczema coxsackium frequently mimics EH. As summarized in recent European pediatric series, Coxsackie A6 disease often shows more polymorphic lesions, less pain, and a predilection for palms, soles, and oral mucosa, though overlap does occur; this justifies confirmatory testing when available [17,18,19]. In our setting, lesional PCR was not available. Blood serology and PCR were therefore obtained as complementary investigations and yielded retrospective findings compatible with HSV-1 infection. Importantly, these results did not influence the initial therapeutic decision, as treatment had already been initiated on clinical grounds according to current recommendations.

A recently published pediatric case by Cheng et al. similarly described eczema herpeticum complicating AD with a favorable response to acyclovir therapy [20]. Similar to our patient, prompt antiviral treatment was associated with rapid clinical improvement. However, our report additionally highlights the challenges posed by the unavailability of lesional PCR and reinforces the importance of initiating treatment based primarily on clinical suspicion rather than awaiting microbiological confirmation.

Management was consistent with current recommendations, with immediate initiation of systemic acyclovir based on clinical suspicion and a low threshold for ophthalmologic referral when ocular involvement is suspected [16,21]. In this case, acyclovir was started, and the child improved rapidly—fever abated within 24 h and lesions progressed to crusting—consistent with cohort data showing that early antiviral therapy reduces hospitalization, mitigates lesion burden, and limits complications [15]. Routine laboratory evaluation showed no evidence of renal or hepatic dysfunction, supporting the safety of oral acyclovir administration and the absence of significant extracutaneous involvement. The child’s age (4 years) placed him outside the highest-risk <1-year group, but he still exhibited other admission predictors described in pediatric series (high fever, systemic symptoms, widespread involvement), underlining the importance of not deferring treatment while awaiting confirmatory tests [15].

A recurrent clinical concern is whether to continue topical corticosteroids during acute EH. Historically avoided, contemporary evidence supports careful re-introduction or continuation after antivirals are underway to control AD inflammation without increasing HSV replication risk [4]. In our patient, prompt antiviral treatment followed by optimization of AD management likely contributed to rapid recovery and restoration of skin barrier function.

Ocular involvement is an EH complication with potential for long-term morbidity. Although our patient presented with mild bilateral conjunctival injection, the literature documents unilateral or bilateral herpetic keratoconjunctivitis requiring prompt systemic and topical antiviral therapy to prevent stromal scarring and permanent visual sequelae [11,13]. Formal ophthalmologic evaluation was not available in our case. However, the absence of photophobia, ocular pain, visual symptoms, or periocular lesions, together with the lack of clinical progression during follow-up, made clinically significant keratitis unlikely.

In summary, this child presented with a painful, monomorphic, rapidly disseminating vesiculopustular eruption superimposed on AD, accompanied by high-grade fever and lack of response to empirical antibiotic therapy. Although lesional PCR was unavailable, the diagnosis was supported by the characteristic clinical findings, and oral acyclovir was initiated immediately without awaiting microbiological confirmation. Blood HSV-1 IgM and PCR subsequently provided retrospective ancillary findings compatible with HSV-1 infection; however, these findings did not influence the clinical diagnosis or therapeutic decision, which had already been established on clinical grounds. This case highlights several practical challenges frequently encountered in routine pediatric care, including initial diagnostic uncertainty, limited access to optimal virological testing, ocular findings at presentation, and the need for rapid clinical decision-making. Together, these elements reinforce the importance of bedside recognition and prompt antiviral treatment in achieving favorable outcomes in eczema herpeticum.

## 4. Limitations

This report has several limitations. First, PCR testing from vesicular lesions, considered the preferred method for virological confirmation, was unavailable. Second, ophthalmologic evaluation could not be performed despite the presence of mild conjunctival injection. Third, additional diagnostic procedures such as Tzanck smear were not performed. Fourth, detailed methodological information regarding the blood PCR assay was not accessible from the hospital laboratory records. Furthermore, the case was reconstructed from the emergency department documentation available in medical records, and no additional laboratory data beyond those reports could be retrieved. Nevertheless, the diagnosis was based primarily on the characteristic clinical presentation, the established risk factors, and the subsequent clinical evolution following initiation of systemic acyclovir. The available blood HSV-1 IgM and PCR results should be interpreted as ancillary retrospective findings of limited diagnostic weight, particularly given the absence of lesional PCR, the lack of paired serological testing, and the unavailability of methodological details for the blood PCR assay. Importantly, the purpose of this case report is not to establish virological confirmation of HSV infection, but rather to document the clinical recognition, diagnostic reasoning, and management of Kaposi’s varicelliform eruption in a real-world pediatric emergency setting.

## 5. Conclusions

Eczema herpeticum should be promptly recognized in children with atopic dermatitis who develop painful monomorphic vesiculopustular lesions associated with fever and rapid dissemination. Immediate systemic acyclovir remains the cornerstone of management and should not be delayed pending laboratory confirmation. Although PCR from vesicular lesions is the preferred diagnostic test when available, clinical judgment remains fundamental, particularly in settings with limited access to specialized investigations. Awareness of potential ocular involvement and optimization of underlying atopic dermatitis after antiviral initiation are also important components of care. While eczema herpeticum is a well-recognized complication of atopic dermatitis, the contribution of this report is not the rarity of the presentation but rather the illustration of clinical decision-making when recommended diagnostic resources are unavailable and management must rely on bedside recognition and prompt therapeutic intervention. This case reinforces the continuing importance of early clinical suspicion and timely antiviral treatment in achieving favorable outcomes in routine pediatric practice.

## Figures and Tables

**Table 1:** Differential diagnosis of disseminated vesiculopustular eruptions in children with AD.

Diagnosis	Characteristic Findings	Findings in Our Patient	Reason Considered Unlikely
Eczema herpeticum	Monomorphic painful vesiculopustules, fever, rapid dissemination	Present	Final diagnosis
Impetiginized eczema	Crusted lesions, bacterial superinfection	AD flare and crusting	Painful monomorphic vesicles, rapid dissemination, and no response to oral antibiotic therapy.
Eczema coxsackium	Oral lesions, palms and soles involvement	Disseminated vesicles	No oral or palmoplantar lesions
Varicella	Lesions in different developmental stages	Disseminated eruption	Monomorphic lesions; fully vaccinated child
Molluscum contagiosum	Umbilicated papules	Previous history	Acute febrile presentation and rapid progression

**Table 2:** Diagnostic investigations performed during the acute episode.

Investigation	Result
Leukocyte count	13,500/µL
Neutrophils	72%
Lymphocytes	20%
Hemoglobin	12.6 g/dL
Platelets	345,000/µL
C-reactive protein (CRP)	32 mg/L
Erythrocyte sedimentation rate (ESR)	28 mm/h
Urea	24 mg/dL
Creatinine	0.42 mg/dL
AST	32 U/L
ALT	25 U/L
Sodium	137 mmol/L
Potassium	4.1 mmol/L
HSV-1 blood PCR	Positive
HSV-1 IgM serology	Positive
Gram stain	Negative
Blood culture	Negative
Tzanck smear	Not performed
Ophthalmologic evaluation	Not available

## Data Availability

The original data presented in the study are included in the article; further inquiries can be directed to the corresponding author.

## Abbreviations

The following abbreviations are used in this manuscript:

EH	Eczema herpeticum
KVE	Kaposi varicelliform eruption
HSV-1	Herpes simplex virus type 1
HSV-2	Herpes simplex virus type 2
AD	Atopic dermatitis
AAAAI	American Academy of Allergy, Asthma & Immunology
ACAAI	American College of Allergy, Asthma & Immunology
AI	Artificial intelligence
